# Housing Conditions and a Challenge with Lipopolysaccharide on the Day of Estrus Can Influence Gene Expression of the Corpus Luteum in Gilts

**DOI:** 10.3390/genes13050769

**Published:** 2022-04-26

**Authors:** Arthur Nery da Silva, Luana Alves, Germana Vizzotto Osowski, Leandro Sabei, Priscila Assis Ferraz, Guilherme Pugliesi, Mariana Groke Marques, Ricardo Zanella, Adroaldo José Zanella

**Affiliations:** 1 Department of Preventive Veterinary Medicine and Animal Health, School of Veterinary Medicine and Animal Science, University of São Paulo, Pirassununga 05508-070, SP, Brazil; arthur.nery@usask.ca(A.N.d.S.); lalves@usp.br (L.A.); germanav.osowski@usp.br (G.V.O.); sabei_le@alumni.usp.br (L.S.); 2 Department of Animal Reproduction, School of Veterinary Medicine and Animal Science, University of São Paulo, Pirassununga 05508-070, SP, Brazil; prisferraz@usp.br (P.A.F.); gpugliesi@usp.br (G.P.); 3 Embrapa Suínos e Aves, Concórdia 89715-899, SC, Brazil; mariana.marques@embrapa.br; 4 Programa de Pós-Graduação em Produção e Sanidade Animal, Instituto Federal Catarinense—IFC, Concórdia 89703-720, SC, Brazil; 5 School of Agronomy and Veterinary Medicine, University of Passo Fundo, Passo Fundo 99052-900, RS, Brazil; ricardozanella@upf.br

**Keywords:** acute stress, angiogenesis, development, LPS, swine, RT-qPCR

## Abstract

The corpus luteum (CL) is a temporary endocrine gland that plays a decisive role in the reproductive physiology of gilts. Recently, it has been suggested that exogenous factors may compromise the normal functioning of the CL. In the present study, we aimed to understand to what extent an acute and systemic challenge with lipopolysaccharide (LPS) on the day of estrus could compromise gene expression of gilts’ CLs housed in different welfare conditions. For this, we housed 42 gilts in three different housing systems: crates, indoor group pens, and outdoor housing. Then, we challenged six females from each group with LPS and eight with saline (SAL) on the day of estrus. After slaughtering the gilts on the fifth day after the challenge, ovaries were collected for gene expression analysis by RT-qPCR. Housing system and LPS challenge did not have a significant interaction for any genes evaluated; thus, their effects were studied separately. We identified significant (*p* < 0.05) downregulation of the angiogenic genes *VEGF* and *FTL1* among LPS-challenged animals. Meanwhile, we also observed upregulation of *HSD3B1* gene among LPS-challenged animals. We found that *STAR* and *LHCGR* genes were differentially expressed depending on the housing system, which indicates that the environment may affect adaptation capabilities. Our results indicate that an acute health challenge on the estrus day alters CL gene expression; however, the role of the housing system remains uncertain.

## 1. Introduction

In swine, the corpus luteum is a transient endocrine gland with a short lifespan, from approximately 12 to 15 days [1], except for the gestation period. The leading hormone secreted by this temporary gland is progesterone (P4) [2,3], which reaches its maximum plasma concentration between days 8 and 9 after ovulation [1,4]. Progesterone not only plays an essential role in the maintenance and success of pregnancy [5] but also causes a direct negative feedback mechanism in the hypothalamus suppressing the secretion of follicle-stimulating hormone (FSH) and luteinizing hormone (LH) [6]. It has been suggested that the inadequate performance of the corpus luteum is one of the principal causes of subfertility and embryonic losses in mammals [2].

There is increasing evidence that the functionality of the corpus luteum can be affected by environmental factors and stress through physiological impairments that involve inflammatory cytokines and androgen excess [6,7]. In addition, valuable efforts have been made to understand what factors affect the development of the corpus luteum during pregnancy [8,9]. Therefore, we hypothesized that the environment in which swine females are housed in the pre-mating period might interfere with the early developmental gene expression on the corpora lutea, which can compromise its maximum progesterone production. These environmental conditions can affect gene expression, as the housing system, in addition to being able to improve the well-being of individuals, can also interfere with the animal’s resilience in the face of a sanitary challenge [10,11].

Infections of the urinary tract of female pigs by environmental bacteria are among the most significant challenges of intensive pig farming [10]. These infections, which can cause systemic diseases, are often caused by Gram-negative bacteria [11]. This class of bacteria has lipopolysaccharides (LPS) in its external membrane, which is responsible for promoting a systemic inflammatory response, including fever, vasodilation, and eicosanoid secretion in their hosts [12]. On the day of insemination, female swine can be susceptible to infections, as not only will the semen be deposited in the female’s cervix or uterus body, but also because there is manipulation with tools and the possibility of introducing environmental bacteria into the reproductive tract of the female pig [11]. Thus, we hypothesize that this breakdown of homeostasis may be associated with reproductive problems, including the establishment of the newly formed corpus luteum.

The study of gene expression by RT-qPCR is recognized as one of the best methods for determining to what extent a gene is being expressed during tissue development and in the face of a health challenge [13].

We hypothesize that the housing system may interfere with the female’s resilience in dealing with a health challenge on the day of estrus, which may compromise the expression of genes related to progesterone synthesis (*STAR, CYP11A, HSD3B1, LHCGR,* and *PGR*), angiogenesis (*VEGF, FLT1,* and *KDR*), inflammation and apoptosis regulation (*IL1B, TNF,* and *IFNG*), and stress response (*HSD11B2, NR3C1,* and *NR3C2*) on the corpora lutea. Therefore, our objective was to evaluate gene expression of the corpora lutea of swine females housed in three housing systems and challenged with LPS—or saline (SAL)—on the estrus day.

## 2. Materials and Methods

Animal experiments were designed and conducted according to the Ethics Principle in Animal Research adopted by the Ethics Committee in the Use of Animals of the School of Veterinary Medicine and Animal Science of the University of São Paulo (CEUAx 9992150121).

### 2.1. Animals and Experimental Design

**Figure 1:**
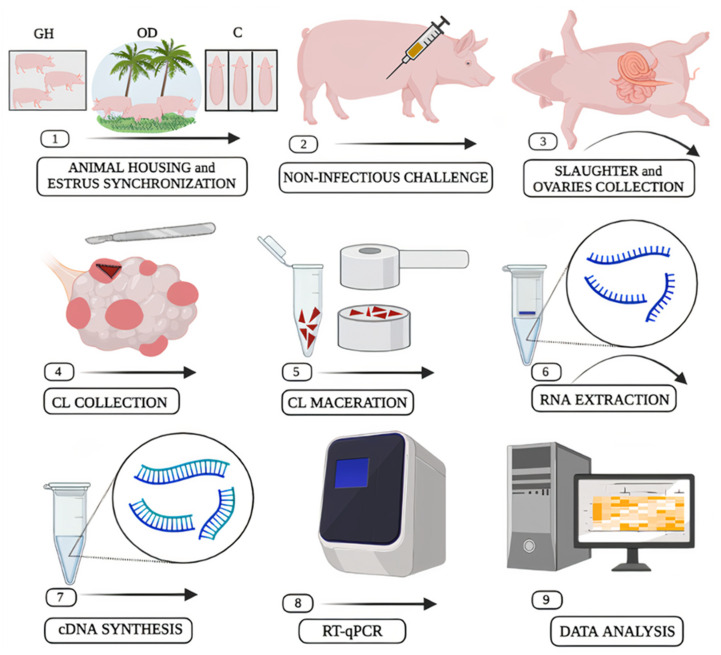
Summarized experimental design.

To determine the effect of LPS on porcine corpora lutea and the environment’s role in coping with this challenge, we used the same animals described in our previous study [14]. Briefly, forty-two gilts from commercial crossbreed lineages participated in the experimental design of this study (Figure 1). All females received water ad libitum and the same commercial nutrition, even females housed in groups because they had individual feeding stalls. In addition, all of them were identified as sexually mature using a boar before the experiment (all of them presented at least one clinical sign of estrus). The animals had their estrous cycle synchronized with Altrenogest (Regumate^®^, MSD Saúde Animal, São Paulo, Brazil) at a dose of 20 mg per animal for 18 days, as recommended by the supplier. Then, the 42 gilts were separated into three groups of 14 animals each: crates (C), indoor group pens (GH), and outside group housing (OD). To enter one of the three housing possibilities, the gilts had their body weight and degree of kinship assessed. Our objective was to keep the group heterogeneous in terms of genetic background and with similar average body scores across all housing groups. The animals were kept throughout the estrous cycle in the specific housing system they were housed. Then, on the day of estrus, six gilts from each housing system received a single dose of 2 µg/kg of LPS (*E. coli* O111:B4, Sigma Aldrich, St. Louis, Missouri, USA) while the other eight received SAL as a control. On the 5th day (~120 h) after the estrus day, all the gilts were slaughtered, and each gilt’s right and left ovaries were collected. Subsequently, the ovaries were immediately frozen in liquid nitrogen and stored at −80 °C.

### 2.2. Corpus Luteum Collection

For macroscopic evaluation and tissue collection, a systematic procedure was organized with liquid nitrogen, which kept the samples frozen throughout the process. Moreover, we used sterile materials for each one of the samples, strictly controlling contamination between samples and by materials. The CL collection was performed collecting fragments with a stab incision with scalpel blade 24 to extract a cone of tissue of the five largest CLs from each ovary. Thus, soon after collecting the biopsies (approximately 0.1 g in total), the fragments were macerated and mixed using a metallic apparatus. During the maceration process, liquid nitrogen was used to preserve the five biopsies of the CLs, which facilitated the tissue maceration procedure. The resulting macerated tissue was stored in cryotubes of 2 mL at −80 °C until RNA extraction.

### 2.3. RNA Extraction and cDNA Synthesis

Approximately 50 ng of macerated CL was used for total RNA extraction, using a standard protocol with TRIzol^®^ (Thermo Fisher Scientific, Waltham, MA, USA) [15]. To check the concentration of the total RNA extracted (A260) and purity (A260/A280), spectrophotometric absorbance was measured in the NanoDrop 2000 (Thermo Fisher Scientific, Waltham, MA, USA). The mean RNA concentration was 0.776 (±0.132) μg/μL and the 260/280 was 2.045 (± 0.043), indicating good RNA quality for the gene expression analysis. Total RNA was treated with DNase I (Life Technologies, Carlsbad, CA, USA) to eliminate eventual contamination with genomic DNA. To finish, the cDNA was synthesized using a High-Capacity cDNA Reverse Transcription Kit (Life Technologies, Carlsbad, CA, USA) according to the manufacturer’s instructions.

The cDNA of each sample was stored at −20 °C until qPCR analysis. Additionally, the final transcriptase reverse reaction was standardized at 1:80, and this cDNA concentration was used as a template for each one of the qPCRs reactions.

### 2.4. Oligonucleotide’s Synthesis

The oligonucleotides *PGR*, *VEGF*, *FLT1*, *KDR*, *STAR*, *CYP11A*, *HSD3B1*, *LHCGR*, *TNF*, and *IFNG* were designed according to gene sequences from Ensembl (http://www.ensembl.org/index.html (accessed on 16 March 2021) and mRNA sequences deposited in GenBank (http://www.ncbi.nlm.nih.gov (accessed on 16 March 2021), avoiding genomic DNA amplification (Supplementary Table S1 [16,17,18]). In addition, the specificity was confirmed through in silico analysis by blasting the sequences of primers against the NCBI database (https://blast.ncbi.nlm.nih.gov/ (accessed on 26 March 2021). For *HSD11B2*, *NR3C1*, *NR3C2*, *IL1B*, and *UBB* genes, previously published primer sets were used [16,17,18].

### 2.5. Quantitative Real-Time PCR

Specific transcripts were quantified using RT-qPCR using PowerUp SYBR Green Master Mix (Life Technologies, Carlsbad, CA, USA) with a final volume of 10 µL per reaction, including a cDNA amount of 2 µL and a primer concentration of 400 nM. The reactions were run in triplicate on a 96-well plate, sealed with a MicroAmp optical adhesive cover (Life Technologies, Carlsbad, CA, USA) before reading in a Step-One Plus Real-Time PCR System (Applied Biosystems, Foster City, CA, USA). The thermocycling profile consisted of 40 cycles of 15 s at 95 °C for denaturation and 12 s at 60 °C for annealing and extension, including a previous activation step of 95 °C for 10 min. The final stage included an analysis of the melting curve, verifying the presence of a single peak in the different PCRs.

### 2.6. Selection of the Housekeeping Gene and Data Normalization

The amplification data were extracted from the Step-One Plus Real-Time PCR System (Applied Biosystems, Foster City, CA, USA), and each sample was analyzed through LinRegPCR (version 2020.2) software [19] for baseline correction, determination of qPCR efficiency, and cycle quantification values per sample. The selection of the reference gene was determined in accordance with Okino et al. [18]., which suggested that *UBB* was the most stable gene for comparative analysis in the corpus luteum of sows. Thus, gene expression of each target gene relative to the housekeeping gene was normalized using the comparative ∆Ct and the fold change due to treatment 2^−∆∆Ct^ [13], using the arithmetic mean (AM) of the ∆Ct values of the SAL challenged group, independently of the housing system. The formula used for normalization was 2^−∆∆Ct^ [13], where ∆∆Ct = [Ct (target gene mRNA) − Ct (UBB)] experimental groups − [AM (Ct (target gene mRNA) − AM (UBB Ct SAL group)].

### 2.7. Statistical Analysis

All data were evaluated by MedCalc©. Independent variables were considered: three housing systems (C, GH, or O) and challenge (LPS or SAL). Dependent variables were considered by fold-change estimates (2^−∆∆Ct^) of gene expression of *STAR, CYP11A, HSD3B1, LHCGR, PGR, VEGF, FLT1, KDR, IL1B, HSD11B2, NR3C1*, and *NR3C2* genes. The *TNF* and *IFNG* oligonucleotides were not amplified in RT-qPCR, nor were they included in the analysis of our study. The outlier analysis was performed using the Tukey test. The Shapiro–Wilk test assessed the normal distribution. Comparisons between independent variables and the interactions were performed using a two-way analysis of variance for ANOVA, with Tukey–Kramer as a post-test. When data were not normally distributed, the Kruskal–Wallis test was used, with Dunn as a post-test. Results were considered significant when *p* < 0.05.

## 3. Results

### 3.1. Morphological Measures of the Ovaries

During the macroscopic evaluation of the 42 ovaries, two ovaries did not present CLs on their surface, and one ovary had only one CL. The ovaries that did not present CLs on their surface were from gilts kept in the crates system (one treated with LPS and the other one with SAL). The ovary with only one CL was from a gilt kept in the outdoor system and treated with LPS. These three samples were removed from our gene expression study because they did not meet our minimum standards of five CLs.

### 3.2. Gene Expression Evaluation on the Corpus Luteum

There was no significant interaction identified between the LPS challenge and the housing system; thus, the effects were studied separately.

**Figure 2:**
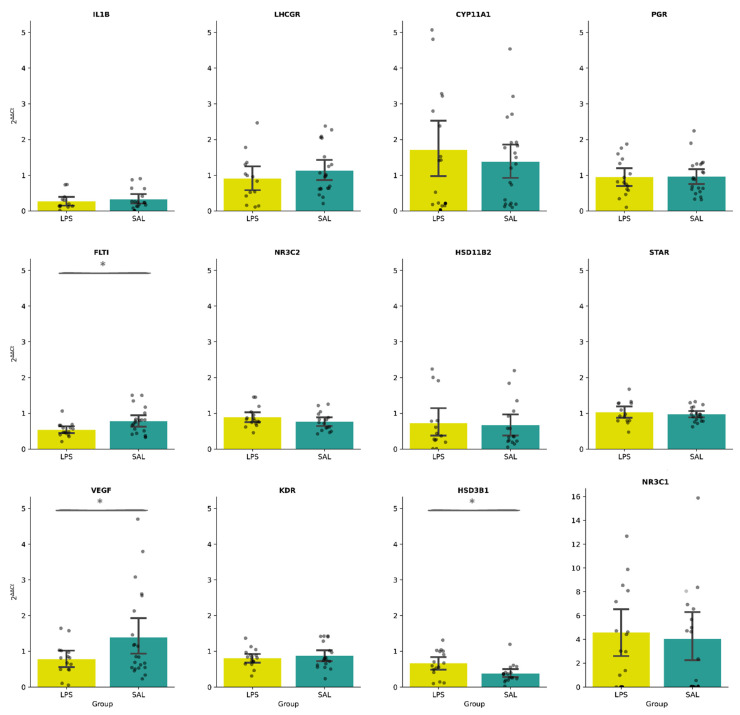
Descriptive analysis of the gene expression of the 12 evaluated genes, considering 2^∆∆Ct^ values. Comparison between LPS (lipopolysaccharide) and SAL (saline solution) groups. Data represent averages ± standard error of the mean (SEM). Significant differences (*p* < 0.05) were represented by an asterisk.

Interestingly, when comparing the animals that received LPS and those that received saline solution (SAL), differences were identified for three genes. For the *FLT1* and *VEGF* genes, animals challenged with LPS experienced significant downregulation of gene expression in relation to the group exposed to SAL (Figure 2; Supplementary Table S2). For the *HSD3B1* gene, animals challenged with LPS experienced significant upregulation of gene expression compared to the group that received SAL (Figure 2; Supplementary Table S2).

**Figure 3:**
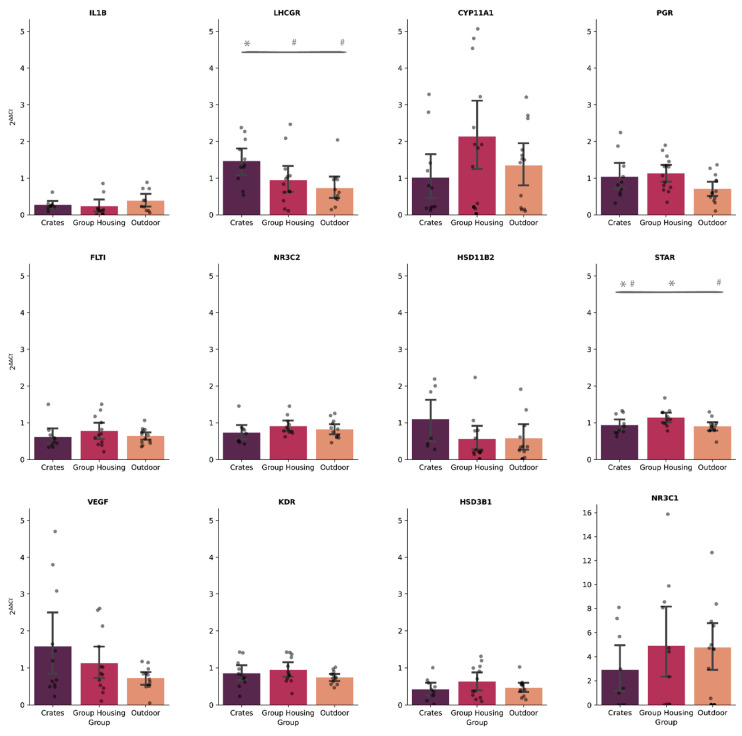
Descriptive analysis of the gene expression of the 12 evaluated genes, considering 2^∆∆Ct^ values. Comparison between housing systems (crates, indoor group pens, or outdoor housing). Data represent averages ± standard error of the mean (SEM). When there were statistically significant differences (*p* < 0.05) between groups, hash (#) or asterisk (*) were used to evidence and facilitate visualization.

When comparing gene expression between housing systems, two differentially expressed genes were identified. *LHCGR* gene was significantly upregulated among animals housed in the crates system, compared to animals from indoor group pens or outdoor housing (Figure 3). Lastly, we detected that the animals from the outdoor housing and indoor group pens systems showed significant differences in the expression of the *STAR* gene, but not among animals housed in crates (Figure 3).

**Figure 4:**
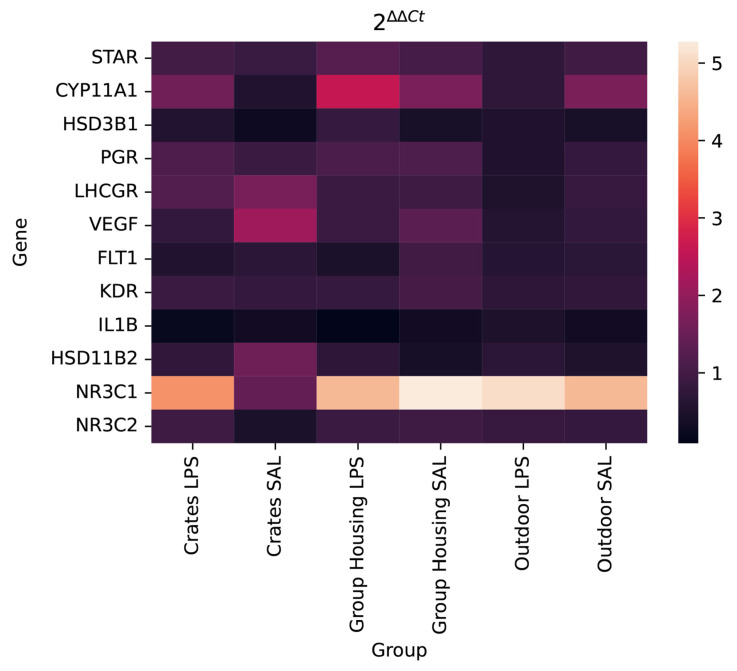
Heatmap of the evaluated genes contrasting with the treatments. The expression values equal to three were designated in red; black means reduced expression and white increased expression. The heatmap was generated by a log transformation due to treatment of the RT-qPCR data.

Overall, the heatmap shows that genes related to the control of progesterone synthesis (*STAR, CYP11A, HSD3B1, LHCGR*, and *PGR*) presented a similar expression pattern. Likewise, genes related to angiogenesis (*FTL1, VEGF*, and *KDR*) and stress response (*IL1B, HSD11B2, NR3C1*, and *NR3C2*) also responded in a modestly similar way, regardless of the housing system or health challenge (LPS/SAL) (Figure 4). As can be seen in the heatmap and Figure 2 and Figure 3, the *NR3C1* gene not only showed a markedly different expression pattern from the other genes (upregulation), but it was also possible to visualize a marked contrast within the group housed in crates. Gilts housed in crates that received SAL showed lower expression of NR3C1 gene than all others, regardless of housing or challenge system (Figure 3).

## 4. Discussion

### 4.1. The Role of Inflammation on Corpus Luteum Gene Expression

Lipopolysaccharide was used for inducing acute inflammatory symptoms in 18 gilts of our experiment. The choice of this systemic inflammatory inducer is justified by its recognized role in terms of breaking the immune homeostasis and impairing the welfare of female pigs [20]. Furthermore, it also simulates one of the biggest medical challenges for females: urinary tract infection by Gram-negative bacteria [21]. It is reported that LPS binds in toll-like receptors 4 (TLR-4) across different cells types [12]. Moreover, previous studies suggested that the activation of these receptors initiates a complex cellular response, resulting in pro-inflammatory mediators such as inflammatory cytokines, reactive oxygen species, and steroid hormones [12]. This state of disrupted homeostasis of the organism generates different outcomes, which include reduced performance [20], neurologic dysfunctions [12], and changes in gene expression [22].

To the best of our knowledge, this report is the first evidence that an in vivo systemic challenge using LPS on the day of estrus can compromise the gene expression of the newly formed corpus luteum of female pigs. Our study identified significant downregulation of the *VEGF* and *FTL1* genes and upregulation of the *HSD3B1* gene in the LPS-challenged group. Notably, we also identified significant differences in the gene expression of the *LHCGR* and *STAR* genes by contrasting their expression depending on the housing system. Interestingly, in the gene expression responses, we did not identify significant interaction between the housing system and the challenge to which the animals were exposed (LPS/SAL). This could suggest that there is no relationship between the variables tested, despite evidence that was demonstrated in our recent study on how outdoor housing of gilts can prevent changes in microbiota in gilts when challenged with LPS, which was observed in stall and group housed animals [14]. This observation, however, may have been influenced by the physiological period the females were experiencing, as high amounts of estrogen were expected to be occurring, and by the limited number of genes studied.

Both the mRNAs transcribed by *VEGF* and *FTL1* in the corpus luteum have been considered the main mitogenic factors for endothelial cells [23,24,25]. In addition, studies have identified differences in the expression of the *VEGF* gene when evaluating animals with different genetic backgrounds [8], or submitted to high doses of steroids [7]. Studies suggest that *VEGF* and *FTL1* play a central role in inducing neovascularization [7,24,25], as well as in the differentiation, maturation, and stabilization of blood vessels in the luteal tissue [8]. Furthermore, it was suggested that animals stressed with the exogenous adrenocorticotropic hormone (ACTH) not only have down expression of the *VEGF* gene but also have genes related to progesterone biosynthesis compromised [7]. Considering these factors, we suggest that the reduced presence of transcripts from the *VEGF* and *FTL1* genes may be involved in reduced nutrition in the corpus luteum and failure to release progesterone from the luteal tissue. This is because, according to Bacci et al. [26], there is a relationship between the reduction in blood vessels, the fading of progesterone, and corpus luteum regression.

Corroborating the findings by Qian et al. [7], which identified downregulation of *VEGF*, *CYP11A1*, and *HSD3B* in the corpora lutea of stress-induced sows by ACTH administration before estrus, our study was also able to identify differential expression in genes related to neovascularization and progesterone synthesis cascade, such as *VEGF*, *FTL1*, and *HD3B1*, but not in *CYP11A1* gene expression. However, it is important to clarify that the source of stress that Qian et al. [7] used was different from ours. The author used repeated acute stress for the stimulation of the adrenal and cortisol secretion. They administered ACTH for 7 days every 8 h before the estrus day, whereas in our study, we used a single dose of LPS on the estrus day. We hypothesize that *VEGF, FTL1,* and *HSD3B1* expression may be more susceptible to downregulation than that of *CYP11A1* gene under stress conditions, or that an extended time period of stress would exacerbate gene expression downregulation.

In a study with stem cells, isoforms of *VEGF* had already been shown to have low secretion when cell cultures were exposed to LPS, compared to a control group exposed to saline. In that study, the researchers identified time dependence concerning exposure to LPS [22]. In addition, the authors argue that a possible mechanism that may be involved in the control of the secretion of *VEGF* isoforms is through the TLR4 when the stem cell cultures are exposed to LPS. We also hypothesize that porcine luteal cells, as evidenced in sheep [27] and cattle [28] luteal cells, may have this receptor on their surface. Furthermore, we suggest that activation of this receptor in pigs—if present—might indirectly compromise the gene expression of other genes involved in the maintenance of the corpus luteum. However, to precisely elucidate this mechanism, we suggest that characterization studies of TLR4 be carried out in the corpus luteum of the porcine model. These studies would be valuable for research in swine reproduction because there is a high relevance of this receptor encoding gene for corpus luteum maintenance, corpus luteum vascularization, and successful maintenance of pregnancy in other species of mammals [27,28].

We also found that the expression of the *HSD3B1* gene in the corpus luteum of LPS-challenged gilts was significantly increased compared to those exposed to SAL. The *HSD3B1* gene encodes a key enzyme that catalyzes the oxidative conversion of pregnenolone to progesterone [29]. This finding is contradictory to the literature, as a decrease in the expression of this gene would be expected [7]; the upregulation of this gene after a LPS challenge may be a compensatory mechanism to produce more progesterone since genes related to vascularization are compromised.

Another novel piece of evidence of our research is that acute stress on the estrus day can have consequences that last for at least ~120 h. Previously, the study by Nordgreen et al. [12] showed that pigs had pro-inflammatory cytokines altered in the central nervous system for about 72 h after challenge, in addition to lower levels of noradrenaline in their hypothalamus, hippocampus, and frontal cortex compared to saline-injected pigs. Thus, our findings suggest that the systemic impairment in the current experiment affected the biological functioning of pigs for longer periods than has been reported previously. Remarkably, the findings that the LPS challenge can compromise both the hypothalamus and ovaries emphasize its importance as a consistent stressor of the hypothalamic–pituitary–gonadal axis in pigs.

### 4.2. The Role of the Housing System on Corpus Luteum Gene Expression

The *LHCGR* and *STAR* genes, which are involved in the progesterone cascade, were found to vary their expression depending on the housing system. *LHCGR* gene, which encodes the receptor for both luteinizing hormone and choriogonadotropin, was upregulated among animals housed in crates when compared to animals housed in indoor group pens or outdoor housing. Remarkably, previous studies have reported that gilts exposed to heat stress for 7 days showed upregulation of the *LHCGR* gene in ovarian tissue [30]. As our experiment was conducted in Brazil, a tropical country, we hypothesized that animals housed outdoors could have an environment with more opportunity for thermoregulation, which helped them to cope with heat stress. However, as we did not identify interactions between the housing systems and the health challenge, these findings are difficult to explain and inferences on their biological meaning may require a larger sample size and a different experimental design.

### 4.3. Our Hypothesis on the Possible Role of Environmental Exposure in Foetal Reprogramming through Corpus Luteum Regulation

Recently, studies in the field of animal welfare and behaviour have reported that piglets born from sows who had suffered chronic stress during pregnancy [31], presented stereotypies [32], and sows subjected to restrictive diets [33] had litters with aggressive behaviour or with lower productive performance. The study by Parada et al. (2021) suggests that lameness in sows, which causes pain and inflammation similar to the LPS challenge, during pregnancy may be associated with fetal reprogramming in-uterus, caused by intergenerational epigenetic mechanisms. Our hypothesis is that the genetic modulation of corpus luteum development may also have an influence on intrinsic aspects of pregnancy, and may also be playing a role in the intrauterine fetal experience. Moreover, we suggest that the segregation of the environmental effect is not only transmitted by epigenetic mechanisms in the germ cells but that there are also molecular mechanisms that control the gene expression of the parents’ glands that support the pregnancy [34]. In other words, we hypothesized that the inefficiency of the CL can compromise the fetus and lameness, a very common challenge encountered in pregnant sows, could be the trigger for the impairment in CL function. This hypothesis becomes clearer when we look from the perspective that the sows with lameness and sows housed in crates—which is associated with poorer welfare—and animals that were challenged with LPS had the greatest impairment of gene expression. However, this hypothesis requires further study to be better elucidated, as we did not perform tissue proteomic analysis or measure reproductive hormones before and after the challenge.

Our study not only sheds light on aspects of fundamental biology but also has relevant implications for animal welfare, management, health, and decision making in swine farms. Together with the findings of Alves et al. (2022), we evidenced that keeping female pigs in production systems with high welfare can mitigate the negative impact of health challenges on reproductive outcomes. This study reinforces the belief that animal welfare is very important for gilts, with measurable impacts on gene expression in the corpus luteum, which in turn can affect reproductive outcomes.

## 5. Conclusions

In conclusion, our study was able to identify that a single dose of LPS on the estrus day can cause down expression of the angiogenic genes *VEGF* and *FTL1* and upregulation of the *HSD3B1* gene in the corpus luteum of gilts up to 120 h post-challenge. Moreover, we were able to identify that the *LHCGR* and *STAR* gene expression can vary depending on the housing system, which may have practical implications. Finally, future studies are necessary to investigate if there is dose-dependence of LPS in in vivo models, and if chronic stress also plays a harmful role in the CL gene expression.

## Data Availability

The datasets generated during this study are available by request to the corresponding authors.

## Supplementary Materials

The following supporting information can be downloaded at: www.mdpi.com/article/10.3390/genes13050769/s1; Table S1: Swine specific oligonucleotide forward (F) and reverse (R) primer sequence (5′-3′), amplicon length of the evaluated genes, and primer efficiency in the standard curve on qPCR. Table S2: Comparisons between independent variables and the interactions, for each of the evaluated genes.
