# Supplementary material for: Housing Conditions and a Challenge with Lipopolysaccharide on the Day of Estrus Can Influence Gene Expression of the Corpus Luteum in Gilts
Source: Genes (Basel). 2022 Apr 26;13(5):769. doi: 10.3390/genes13050769 (PMC9141224; doi:10.3390/genes13050769)
Supplement: Supplementary file 1 [file genes-13-00769-s001.zip › genes-1656549-supplementary.pdf]

Table S1. Swine specific oligonucleotide forward (F) and reverse (R) primer sequence (5′-3′), amplicon length of the evaluated genes, and primer efficiency in the standard curve on qPCR.

Target name	GenBank ID	Primer (5′-3′)	Amplicon	Efficiency (%)	Melting temperature	Reference
PGR	NM_001166488.1	F: 5′AACACCAAACCCGACACTTC 3′<br>R: 5′ CGAAAACCTGGCAGTGACTT 3′	107 bp	89.80	80.22	This study
VEGF	X81380	F: 5′ CGAAGTGGTGAAGTTCATGG 3′<br>R: 5′ ACACAGGACGGCTTGAAGAT 3′	120 bp	99.07	83.96	This study
FLT1	AJ245445.1	F: 5′ ACCCCGGAAATCTATCAGATCA 3′<br>R: 5′ GGTCGCCTAGTTTTTCCACAAG 3′	94 bp	87.97	79.63	This study
KDR	AJ245446.1	F: 5′ CTCAGCAGGATGGCAAAGACTA 3′<br>R: 5′ GGGGTCACACACTTCCTCTTCT 3′	128 bp	86.90	81.56	This study
STAR	NM213755	F: 5′CAGACTTTGGAGAGATGCCTGA 3′<br>R: 5′ ATCCCTTGAGGTCAATGCTGAG 3′	138 bp	90.10	86.48	This study
CYP11A1	NM_214427.1	F: 5′ CCTGCCAAGACATTGGTACAAG 3′<br>R: 3′ AGGTCCCTTTCTTTACCCAACC 3′	113 bp	86.41	84.39	This study
HSD3B1	NM_001004049.1	F: 5′ TGGTCATCCACACTGCCTCTAT 3′<br>R: 5′ GGAGCTGGGTACCTTTCACATT 3′	90 bp	91.11	83.2	This study
LHCGR	XM_021085888.1	F: 5′ CATAACCACCGTACCAGCAA 3′<br>R: 5′ TTCAGCTCCAGGGAAATCAG 3′	135 bp	98.80	78.13	This study
HSD11B2	AF414125	F:5′ GCGAAAGCTTCCCACTGAAC 3′<br>R: 5′ AGGGTCTGTTTGGGCTCATG 3′	59 bp	102.63	83.2	[1]

NR3C1	AF141371	F: 5′ GATCATGACCGCACTCAACATG 3′<br>R: 5′ TTGCCTTTGCCCATTTCAC 3′	68 bp	97.11	82.31	[1]
NR3C2	XM_013978840.2	F: 5′ TTGCCTTGAGCTGGAGATCG 3′<br>R: 5′ GAACTGCAGGCTGATCTGGT 3′	143 bp	106.17	82.01	[1]
TNF	NM_214022.1	F: 5' GCCCTTCCACCAACGTTTTC 3'<br>R: 5' CAAGGGCTCTTGATGGCAGA 3'	97 bp	–	–	This study
IFNG	NM_213948.1	F: 5′ GCGCAAAGCCATCAGTGAAC 3′<br>R: 5′ GCTCTCTGGCCTTGGAACAT 3′	105 bp	–	–	This study
IL1B	XM_021085847.1	F: 5′ TTTGAAGAAGAGCCCATCATCC 3′<br>R: 5′ CCAGCCAGCACTAGAGATTTG 3′	119 bp	97.98	81.41	[2]
UBB	U72496.1	F: 5′ ACCAGCAGCGTCTGATTTTT 3′<br>R: 5′ CAAGTGCAGGGTGGACTCTT 3′	92 bp	100.03	82.61	[3]

References

1. Poletto, R.; Steibel, J.P.; Siegford, J.M.; Zanella, A.J. Effects of early weaning and social isolation on the expression of glucocorticoid and mineralocorticoid receptor and 11β-hydroxysteroid dehydrogenase 1 and 2 mRNAs in the frontal cortex and hippocampus of piglets. *Brain Res.* **2006**, *1067*, 36–42, doi:10.1016/j.brainres.2005.10.001.

2. Makowczenko, K.G.; Jastrzebski, J.P.; Szeszko, K.; Smolinska, N.; Paukszto, L.; Dobrzyn, K.; Kiezun, M.; Rytelewska, E.; Kaminska, B.; Kaminski, T. Transcription analysis of the chemerin impact on gene expression profile in the luteal cells of gilts. *Genes (Basel).* **2020**, *11*, 1–18, doi:10.3390/genes11060651.

3. Okino, C.H.; Ibelli, A.M.G.; Brentano, L.; Zanella, R.; Groke Marques, M. Evaluation of candidate reference genes for investigation of the uterine tissue and corpus luteum of pigs on day 6 after artificial insemination. *Anim. Reprod. Sci.* **2018**, *198*, 202–209, doi:10.1016/j.anireprosci.2018.09.020.

Table S2. Comparisons between independent variables and the interactions, for each of the evaluated genes.

Target gene	Housing	Challenge	Housing*Challenge
IL1B	0.261	0.543	0.221
LHCGR	0.027*	0.187	0.661
CYP11A1	0.108	0.473	0.141
PGR	0.067	0.897	0.492
FLT1	0.598	0.024*	0.144
NR3C2	0.256	0.214	0.081
HSD11B2	0.162	0.835	0.238
STAR	0.015*	0.577	0.060
VEGF	0.167	0.044*	0.314
KDR	0.292	0.487	0.513
NR3C1	0.479	0.602	0.687
HSD3B1	0.251	0.013*	0.493

*Significant differences ($p<0.05$) were represented by an asterisk.
